# FRC transplantation restores lymph node conduit defects in laminin **α**4–deficient mice

**DOI:** 10.1172/jci.insight.167816

**Published:** 2023-04-24

**Authors:** Lushen Li, Long Wu, Allison Kensiski, Jing Zhao, Marina W. Shirkey, Yang Song, Wenji Piao, Tianshu Zhang, Zhongcheng Mei, Samuel J. Gavzy, Bing Ma, Vikas Saxena, Young S. Lee, Yanbao Xiong, Xiaofei Li, Xiaoxuan Fan, Reza Abdi, Jonathan S. Bromberg

**Affiliations:** 1 Department of Surgery, and; 2 Center for Vascular and Inflammatory Diseases, University of Maryland School of Medicine, Baltimore, Maryland, USA.; 3 Transplantation Research Center, Renal Division, Brigham and Women’s Hospital, Harvard Medical School, Boston, Massachusetts, USA.; 4 Institute for Genome Sciences, University of Maryland School of Medicine, Baltimore, Maryland, USA.; 5 Flow Cytometry Shared Service, Greenebaum Comprehensive Cancer Center, Baltimore, Maryland, USA.

**Keywords:** Immunology, Laminin, Tolerance

## Abstract

Fibroblastic reticular cells (FRCs) play important roles in tolerance by producing laminin α4 (Lama4) and altering lymph node (LN) structure and function. The present study revealed the specific roles of extracellular matrix Lama4 in regulating LN conduits using FRC-specific KO mouse strains. FRC-derived Lama4 maintained conduit fiber integrity, as its depletion altered conduit morphology and structure and reduced homeostatic conduit flow. Lama4 regulated the lymphotoxin β receptor (LTβR) pathway, which is critical for conduit and LN integrity. Depleting LTβR in FRCs further reduced conduits and impaired reticular fibers. Lama4 was indispensable for FRC generation and survival, as FRCs lacking Lama4 displayed reduced proliferation but upregulated senescence and apoptosis. During acute immunization, FRC Lama4 deficiency increased antigen flow through conduits. Importantly, adoptive transfer of WT FRCs to FRC Lama4–deficient mice rescued conduit structure, ameliorated Treg and chemokine distribution, and restored transplant allograft acceptance, which were all impaired by FRC Lama4 depletion. Single-cell RNA sequencing analysis of LN stromal cells indicated that the laminin and collagen signaling pathways linked crosstalk among FRC subsets and endothelial cells. This study demonstrated that FRC Lama4 is responsible for maintaining conduits by FRCs and can be harnessed to potentiate FRC-based immunomodulation.

## Introduction

Lymph nodes (LNs) serve as the primary sites for initiating immune responses through choreographing crosstalk among immune cells, including T and B cells, and dendritic cells (DCs). The conduits bridge afferent lymphatic vessels and efferent lymphatics. Conduits channel small molecules (<70 kDa), such as antigen and chemokines, from the subcapsular sinus to the inner cortical parenchyma, thereby ensuring antigen sensing (1–6). DCs embedded in the conduits capture soluble molecules in transit (7), facilitating LNs to sense necessary immune signals emanating from inflamed tissues (8). Antigen and chemokine transport through conduits plays an important role in adaptive immune responses (7).

As a hallmark of LN microanatomy, the conduit network is widely distributed through the LN T cell zones (T zones) and B follicles, which are enriched with fibroblastic reticular cells (FRCs) and follicular DCs (FDCs), respectively (9). In LN T zones, FRCs encase and deposit various extracellular matrix (ECM) components to conduits through polarized microtubules (1, 5, 10–13). These ECM components include ER-TR7 (collagen VI), laminins, nidogen-1, fibronectin, and collagens I, IV, and XIV (1, 10, 14–16). Collagens I and IV and ER-TR7 assemble fibrillar chains that wrap the conduits and provide tensile strength. The core of the conduit is primarily composed of collagen XIV, which crosslinks collagen I and limits fibril diameter, preventing lateral binding of adjacent fibrils (14, 17). Relative to the T zones, B follicles have sparse and poorly branched conduits. The B follicle conduits are ensheathed by FDCs, with similar diameters and particle size permissiveness (<70 kDa) as T zone conduits (9, 18).

Laminins are functional ECM components in the basement membrane (19). We previously showed that laminin α4 (Lama4) and α5 (Lama5) correlated with tolerance and immunity, respectively (19–22). Another study showed that Lama4 regulates the structural integrity of newly formed capillaries (23). Newborn Lama4-deficient mice displayed delayed deposition of collagen IV and nidogen into capillary basement membranes, with discontinuities in the lamina densa (23). In LNs, Lama4 and Lama5 are the predominant isoforms in the conduit basement membranes (1, 7). Lama4 and Lama5 are expressed in the outer layer of the conduit that is in direct contact with FRCs (7). LNs lacking FRC Lama4 have a defective FRC network (24). Hence, we hypothesized that FRC-derived laminin 4 is involved in maintaining the conduit system.

The present study investigated the function of FRC-derived Lama4 in maintaining LN conduits and the mechanistic implications for its role in transplant tolerance. Using different conditional KO mouse strains, including CCL19/iDTR, FRC-Lama4-KO, FRC-Lama5-KO, and FRC-LTβR-KO, we demonstrated the molecular requirements of FRCs for LN conduit development and homeostasis. FRC laminins maintain intact LN collagen fibers and conduits, and regulate FRC proliferation, senescence, and apoptosis. Depleting laminins in FRCs reduced the expression of lymphotoxin β receptor (LTβR), the deficiency of which also resulted in defective conduits. FRC cell therapy in which WT FRCs were transferred into Lama4-KO mice rescued conduit and fibroblastic fiber structure and function, and restored allograft acceptance. Overall, this study unveiled mechanisms underlying laminin-regulated FRC homeostasis and conduit structure and function.

## Results

### LN FRCs support conduits.

**Figure 1:**
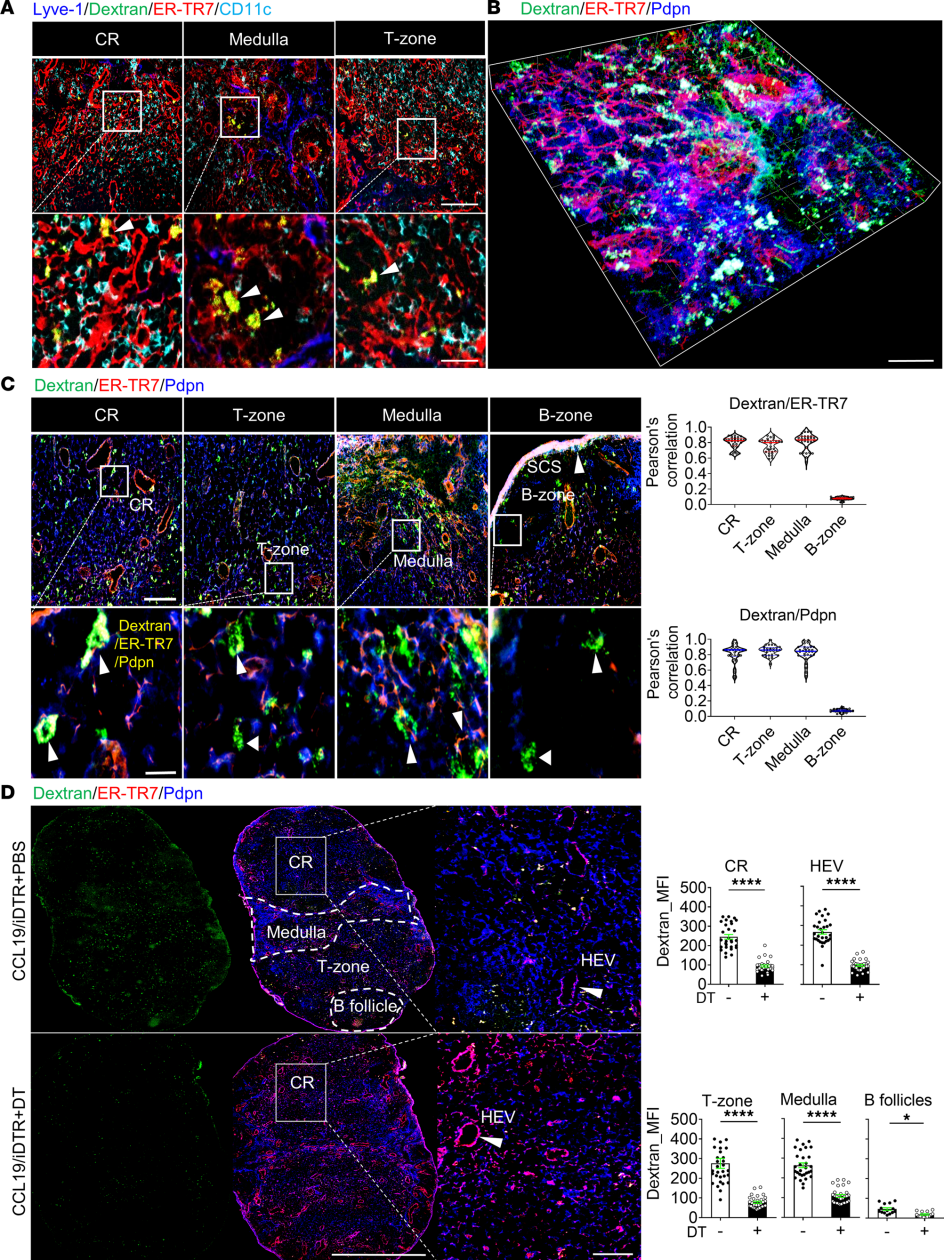
Lymph node FRCs support conduits. C57BL/6 WT mice received 2.5 μg dextran-FITC (40 kDa) s.c. at the tail base. Five minutes after injection, the inguinal draining lymph nodes (dLNs) were collected. (**A**) Conduits colocalized with ER-TR7 but not with Lyve-1 and conventional DCs (cDCs). Arrows point to yellow resulting from the overlap of dextran-FITC (green) and ER-TR7 (red) signals. LN cryosections (6 μm) were stained for Lyve-1, ER-TR7, and CD11c. Original magnification, ×20. Scale bars: 100 μm (top) and 25 μm (bottom). (**B**) 3D confocal immunofluorescence microscopy with 50-μm cryosection stained for ER-TR7 and Pdpn. Representative image shows the CR region. Scale bar: 30 μm. (**C**) Conduits colocalized with ER-TR7 and Pdpn. Left: Sections (6 μm) stained for ER-TR7 and Pdpn. Original magnification, ×20. Scale bars: 100 μm (top) and 25 μm (bottom). Arrows point to conduits that are colocalized with ER-TR7 and Pdpn. Right: Pearson’s correlation for colocalization of dextran-FITC with ER-TR7 or Pdpn in the CR, T zone, medulla, and B zone. (**D**) Depleting LN FRCs impaired the conduit system. CCL19/iDTR mice received diphtheria toxin (DT) (100 ng/day i.p. for 5 days) for FRC depletion. Mice were then injected s.c. with 2.5 μg dextran-FITC (40 kDa) and dLNs harvested after 5 minutes. Left: Whole-mount scanning image s (×20) of 6-μm LN cryosections stained for ER-TR7 and Pdpn. Scale bars: 500 μm (left) and 50 μm (enlarged). Arrows point to high endothelial venules (HEVs). Right: Quantification of dextran-FITC intensity in the CR, T zone, medulla, B follicles, and around HEVs; 3 mice/group, 5 LNs/mouse, 3 sections/LN, 3–5 fields/section. Data presented as mean ± SEM. *P < 0.05, ****P < 0.0001 by 2-tailed Student’s *t* test for 2-group comparisons.

We visualized the LN conduit system by injecting mice subcutaneously (s.c.) with the soluble tracer dextran-FITC (40 kDa). Based on the validated FRC markers podoplanin (Pdpn) and ER-TR7 (25), the immunofluorescence images of draining LN (dLN) cryosections showed that conduits colocalized with ER-TR7^+^ FRCs but did not contact Lyve-1^+^ lymphatic endothelial cells (LECs) or CD11c^+^ DCs (Figure 1A). We next observed conduits more closely using 3-dimensional (3D) confocal microscopy. In the cortical ridge (CR), T zone, and medulla, dextran-FITC indicated conduits were distributed along ER-TR7^+^Pdpn^+^ FRC fibers (Figure 1, B and C). In comparison, the B follicles had smaller Pdpn^+^ FRC fibers and sparser conduits (Figure 1C). Pearson’s correlation values showed that conduits highly colocalized with ER-TR7 and Pdpn in the CR, T zone, and medulla, but not in the B follicles (Figure 1C). These results indicate that LN conduits are widely spread throughout most LN compartments and are intimately associated with FRCs in the Pdpn^+^ regions.

To determine the functional impact of FRCs on conduits, we used CCL19/iDTR mice, which allow rapid inducible depletion of CCL19^+^ FRCs after injection with diphtheria toxin (DT). Whole-mount scanning immunofluorescence images confirmed that conduits were widely distributed in the LNs prior to CCL19^+^ FRC depletion (Figure 1D). FRC depletion significantly reduced the number of conduits in almost all LN regions, including the CR, around high endothelial venules (HEVs), T zones, medulla, and B follicles (Figure 1D). Taken together, these results suggest that FRCs support an intact LN conduit system. Since depleting FRCs leads to rapid LN collapse (24), complete depletion of FRCs is too destructive and not specific enough to dissect out the regulatory factors. Thus, we next utilized additional FRC conditional KO mouse strains to identify specific molecules accounting for the FRC-regulated conduit system.

### FRC Lama4 maintains intact collagen fibers and conduits.

To reveal the specific role of FRC Lama4 and FRC Lama5 in conduit development, we conditionally depleted laminins in FRCs, using the *Pdgfrb* promoter to drive Cre-lox expression in FRCs. Immunofluorescence imaging showed a high extent of colocalization of PDGFRβ and ER-TR7 (Supplemental Figure 1A; supplemental material available online with this article; https://doi.org/10.1172/jci.insight.167816DS1). This was commensurate with single-cell RNA sequencing (scRNA-seq) data showing that *Pdgfrb* expression highly overlapped with collagen type VI gene expression in various FRC subsets (Supplemental Figure 1, B and C). Recently, the ER-TR7 epitope was identified to be collagen type VI (15). *Pdgfrb*-Cre^+/–^ mice were crossed with *Lama4^fl/fl^* and *Lama5^fl/fl^* mice to obtain *Pdgfrb*-Cre^+/–^ × *Lama4^fl/fl^* (FRC-Lama4-KO) and *Pdgfrb*-Cre^+/–^ × *Lama5^fl/fl^* (FRC-Lama5-KO) strains. The KO strains were healthy, with normal LN cellularity relative to wild-type (WT) littermates (21, 24). Both FRC-Lama4-KO and FRC-Lama5-KO LNs retained intact fibroblastic networks (Supplemental Figure 1A). We previously confirmed that Lama4 and Lama5 were specifically depleted in FRCs in FRC-Lama4-KO and FRC-Lama5-KO mice, respectively (24). These results suggest that *Pdgfrb* is an appropriate gene promoter for FRC-conditional depletion of Lama4 and Lama5.

**Figure 2:**
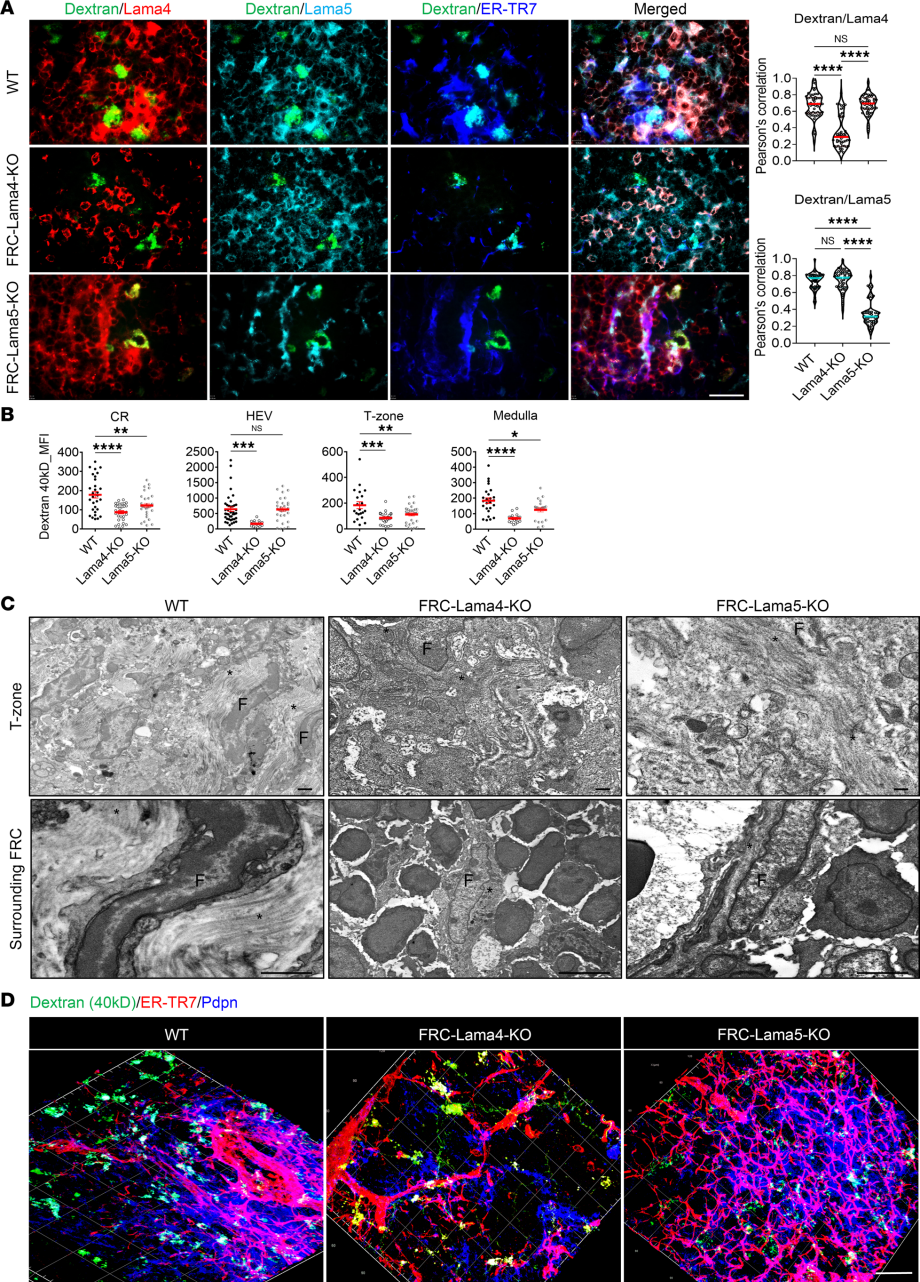
FRC Lama4 is necessary for intact collagen fibers and conduits. (**A**) WT and KO mice received 2.5 μg dextran-FITC (40 kDa) s.c. The draining LNs were harvested after 2 minutes. LN cryosections (6 μm) were stained for Lama4, Lama5, and ER-TR7. Left: Fluorescence images (×20) of LN T cell zone. Scale bar: 100 μm. Right: Pearson’s correlation for colocalization of dextran-FITC with Lama4 and Lama5. (**B**) Quantification of dextran-FITC (40 kDa) in the CR, around HEVs, T zone, and medulla. (**C**) TEM images of WT, FRC-Lama4-KO, and FRC-Lama5-KO LNs (longitudinal section). Scale bars: 500 nm. Asterisks, collagen fibers; F, FRCs. (**D**) 3D confocal immunofluorescence image of 50-μm cryosections stained for ER-TR7 and Pdpn. Scale bar: 30 μm. Data in **A**–**D** are representative of 3 independent experiments; 3 mice/group, 5 LNs/mouse, 3 sections/LN, 3–5 fields/section. Data presented as mean ± SEM. *P < 0.05; **P < 0.01; ***P < 0.001; ****P < 0.0001 by 1-way ANOVA with Tukey’s multiple-comparison test (**A** and **B**).

Lama4 or Lama5 proteins surrounding conduits were clearly reduced in their respective KO LNs (Figure 2A). The conduits in WT LNs were intact and completely filled with dextran-FITC (40 kDa). In contrast, the conduits in FRC-Lama4-KO and FRC-Lama5-KO LNs were partially filled with dextran-FITC, with reduced FITC signals relative to the WT controls. Dextran-FITC intensity was lower in FRC-Lama4-KO in the CR, T zone, medulla, and around HEVs compared with WT (Figure 2B). Depleting FRC Lama5 also reduced conduits relative to WT, but not as much as that observed in FRC Lama4–deficient LNs (Figure 2B). No differences were observed between groups in the B follicles (data not shown). Under transmission electron microscopy (TEM), both FRC-Lama4-KO and FRC-Lama5-KO LNs displayed disorganized T zones relative to WT (Figure 2C). The collagen fibers surrounding FRCs in both types of KO LNs displayed fragmented and disorganized shapes, decreased numbers and decreased thickness, with more severe impairment in FRC-Lama4-KO LNs (Figure 2C). 3D confocal microscopy confirmed that both FRC-Lama4-KO and FRC-Lama5-KO LNs displayed lower dextran-FITC intensity than WT (Figure 2D). WT and FRC-Lama5-KO LNs had normal-appearing ER-TR7^+^ fibroblastic fibers (Figure 2D). In contrast, the FRC-Lama4-KO LNs had disorganized and decreased ER-TR7^+^ fibroblastic fibers (Figure 2D). Hence, FRC-derived Lama4 and Lama5 contributed to the LN structure and conduit system but to different extents. These results indicated that depletion of FRC-Lama4 in particular significantly interfered with the FRC and conduit structure and function. Next, we sought to reveal mechanisms of how Lama4 contributes to FRC and conduit integrity, relate this to the effects of Lama4 on alloreactivity, and ameliorate the defects in FRC-Lama4-KO mice.

### FRC Lama4 affects the intact FRC network and conduits through LTβR signaling.

**Figure 3:**
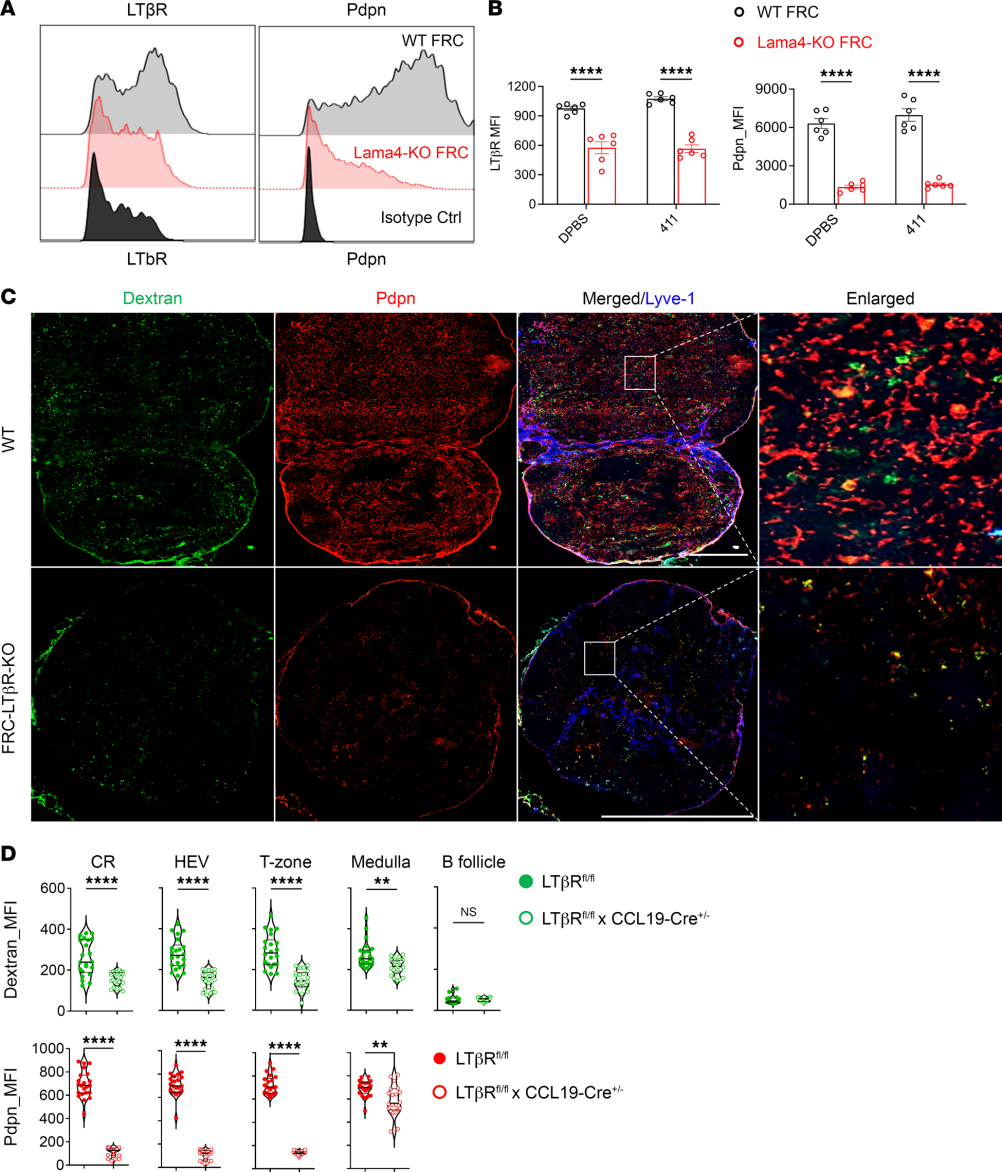
LTβR signaling is required for laminin-regulated conduits. (**A**) LTβR and Pdpn expression on cultured WT and Lama4-KO FRC primary cells after 4 passages. (**B**) Summary of LTβR and Pdpn intensity on WT and Lama4-KO FRC primary cells cocultured with DPBS or heterotrimeric laminin α1β1γ1 (laminin 411) proteins. (**C**) WT and FRC-LTβR-KO mice received 2.5 μg dextran-FITC (40 kDa) s.c. Draining LNs were collected 5 minutes after injection. Whole-mount scanning immunofluorescence images (×20) of 6-μm cryosections stained for Pdpn and Lyve-1. Scale bars: 500 μm. (**D**) Quantification of dextran-FITC (40 kDa) and Pdpn intensity in LN CR, around HEVs, T zone, medulla, and B follicles. Representative of 3 independent experiments; 3 mice/group, 5 LNs/mouse, 3 sections/LN, 3–5 fields/section. Data presented as mean ± SEM. **P < 0.01; ****P < 0.0001 by 2-way ANOVA with Tukey’s multiple-comparison test (**B**) or unpaired, 2-tailed Student’s *t* test for 2-group comparisons (**D**).

LTβR and Pdpn signaling are required for FRC and LN development and dictate FRC phenotype (13, 26). We next explored the contribution of LTβR in laminin-regulated FRCs and conduits. Compared with WT FRCs, Lama4-KO FRCs had markedly lower expression of LTβR and Pdpn (Figure 3, A and B). Supplementing heterotrimeric protein laminin α4β1γ1 in vitro did not reverse these defects (Figure 3B), indicating that endogenous FRC Lama4 was indispensable for normal LTβR and Pdpn expression. Since LTβR signaling contributes to LN development and angiogenesis (13, 26), we explored the influence of defective FRC LTβR on the conduit system. Dextran-FITC was injected into *Ccl19*-Cre^+/–^ × *Ltbr^fl/fl^* (FRC-LTβR-KO) mice, which have conditional depletion of LTβR in FRCs. Compared with WT, depleting FRC LTβR diminished Pdpn, with a collapsed fibroblastic network in LNs (Figure 3C), indicating that FRC LTβR played an important role in supporting LN architecture. Moreover, compared with WT, FRC-LTβR-KO LNs had decreased conduits in the CR, T zone, medulla, and around HEVs (Figure 3D). No difference was observed in the B follicles (Figure 3D). Taken together, these results indicated that LTβR signaling accounted at least partly for Lama4-regulated FRC integrity and conduit generation.

### FRC Lama4 regulates FRC proliferation and survival.

**Figure 4:**
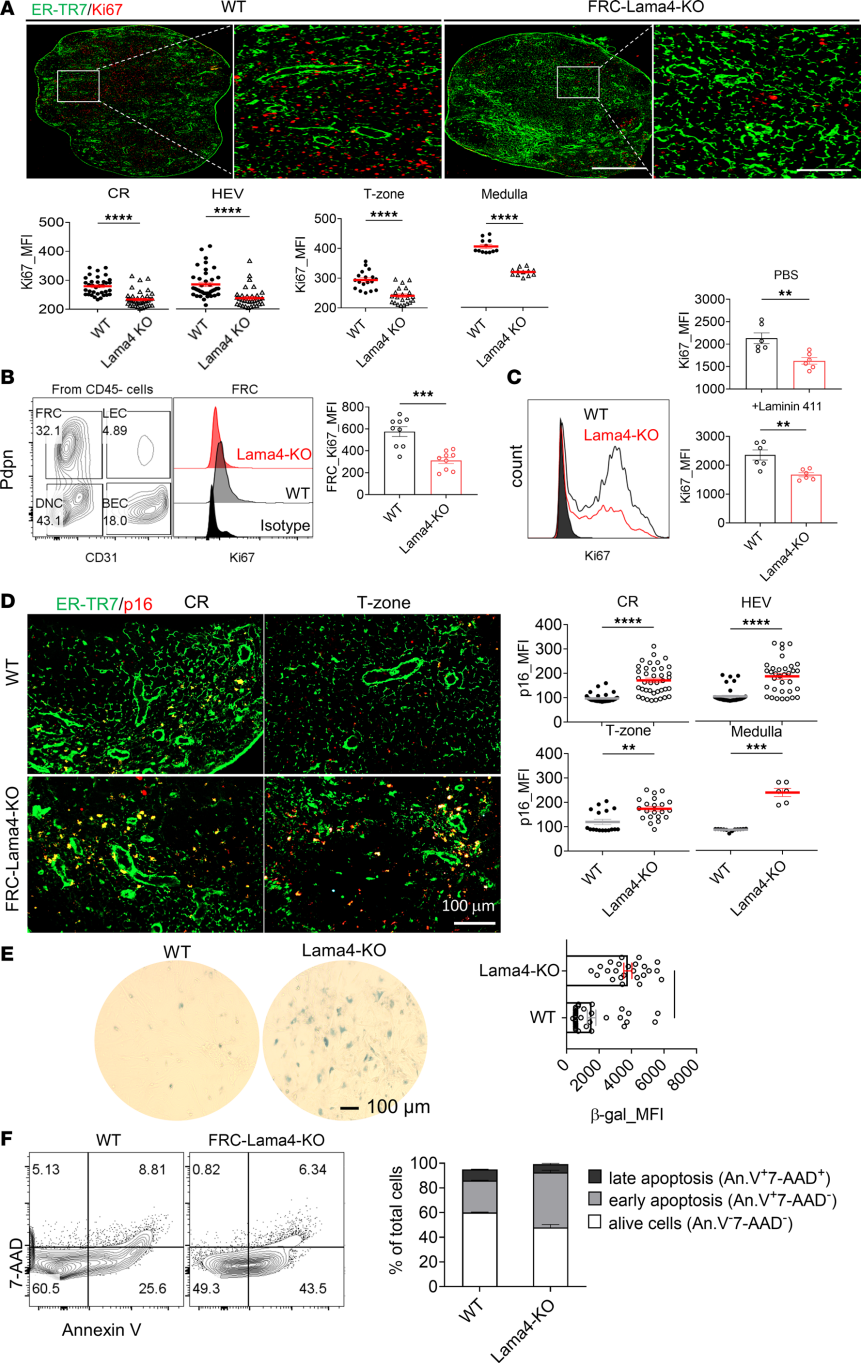
FRC Lama4 regulates FRC proliferation and survival. (**A**) Fluorescence images (×20) of WT and FRC-Lama4-KO LN cryosections stained for ER-TR7 and Ki67. Ki67 intensity was quantified in the CR, around HEVs, T zone, and medulla. Scale bars: 500 μm (left) and 100 μm (right). (**B**) Ki67 expression in freshly isolated FRCs from WT and FRC-Lama4-KO LNs. (**C**) Left: Ki67 intensity in cultured primary WT and Lama4-KO FRCs. Right: Ki67 intensity in primary WT FRCs and Lama4-KO-FRCs cocultured with PBS or heterotrimeric laminin α1β1γ1 (laminin 411) protein. (**D**) Representative fluorescence images (×20) of LN cryosections from WT and FRC-Lama4-KO mice stained for ER-TR7 and p16. p16 intensity quantification in the CR, around HEVs, T zone, and medulla. Scale bar: 100 μm. (**E**) Primary FRCs (passage 4) were isolated from WT and FRC-Lama4-KO mice and stained for β-galactosidase 1 day after subculture. Images (×20) quantified for intensity of β-galactosidase. (**F**) Primary FRCs were isolated from WT and FRC-Lama4-KO mice and stained with Annexin V and 7-AAD. Data in **A**–**F** are representative of 3 independent experiments; 3 mice/group, 5 LNs/mouse, 3 sections/LN, 3–5 fields/section. Data are presented as mean ± SEM. **P < 0.01; ***P < 0.001; ****P < 0.0001 by 2-tailed Student’s *t* test for single variable differences.

The defective FRC networks with decreased Pdpn density in Lama4-KO LNs suggest a regulatory role for Lama4 in FRC proliferation and survival. For in vivo evaluation of FRC proliferation, KO and WT LN cryosections were stained for Ki67. FRC-Lama4-KO LNs expressed significantly lower levels of Ki67 in the FRC-supported CR, HEV surroundings, T zone, and medulla (Figure 4A). To identify the main contributor to these differences, Ki67 expression was assessed in freshly isolated LN cells. Freshly isolated primary Lama4-KO FRCs had decreased Ki67 expression (Figures 4B), indicating downregulated proliferation compared with WT. In contrast, CD4^+^ and CD8^+^ T cells, conventional DCs (cDCs), and plasmacytoid DCs (pDCs) and showed no differences between WT and FRC-Lama4-KO groups (Supplemental Figure 2). Thus, the differences in proliferation observed by histology were due mostly to changes in FRCs. Similarly, ex vivo–expanded primary Lama4-KO FRCs had lower proliferation (Figure 4C). Coating heterotrimeric laminin α4β1γ1 on primary FRC culture plates failed to reverse the changes caused by genetic laminin depletion (Figure 4C). Overall, these results demonstrated that endogenous FRC-derived Lama4 is an intrinsic regulator of FRC proliferation.

We next examined the effects of laminins on FRC senescence using the senescence marker p16. Immunofluorescence images of LNs showed that p16 was higher in the FRC-enriched regions, including the CR, around HEVs, T zone, and medulla in Lama4-KO LNs compared with WT (Figure 4D). Moreover, the p16 signal was highly colocalized with the ER-TR7^+^ FRC fibers (Figure 4D), suggesting the p16 signal is attributable to stromal cells. We measured p16 expression in freshly isolated LN CD4^+^ and CD8^+^ T cells, B cells, cDCs, and pDCs. In these cell types, no pronounced differences were detected among the Lama4-KO and WT groups (Supplemental Figure 3), indicating these cells did not contribute to the p16 differences among the different strains. In vitro, Lama4-KO primary FRCs expressed more β-galactosidase, another marker of senescence, than WT (Figure 4E). These results again indicated that primary FRCs lacking Lama4 were more prone to senescence than WT. We then compared apoptosis of WT and Lama4-KO FRCs by staining with Annexin V and 7-AAD. The Lama4-KO FRCs had fewer viable cells (Annexin V^–^7-AAD^–^) and more early apoptotic (Annexin V^+^7-AAD^–^) populations (Figure 4F). This indicated that FRCs lacking Lama4 were prone to apoptosis. Collectively, these results demonstrated that Lama4 is critical for FRC survival, as FRCs lacking Lama4 have downregulated proliferation but upregulated senescence and apoptosis.

### FRC Lama4 regulates inflammatory conduit flow and antigen uptake.

**Figure 5:**
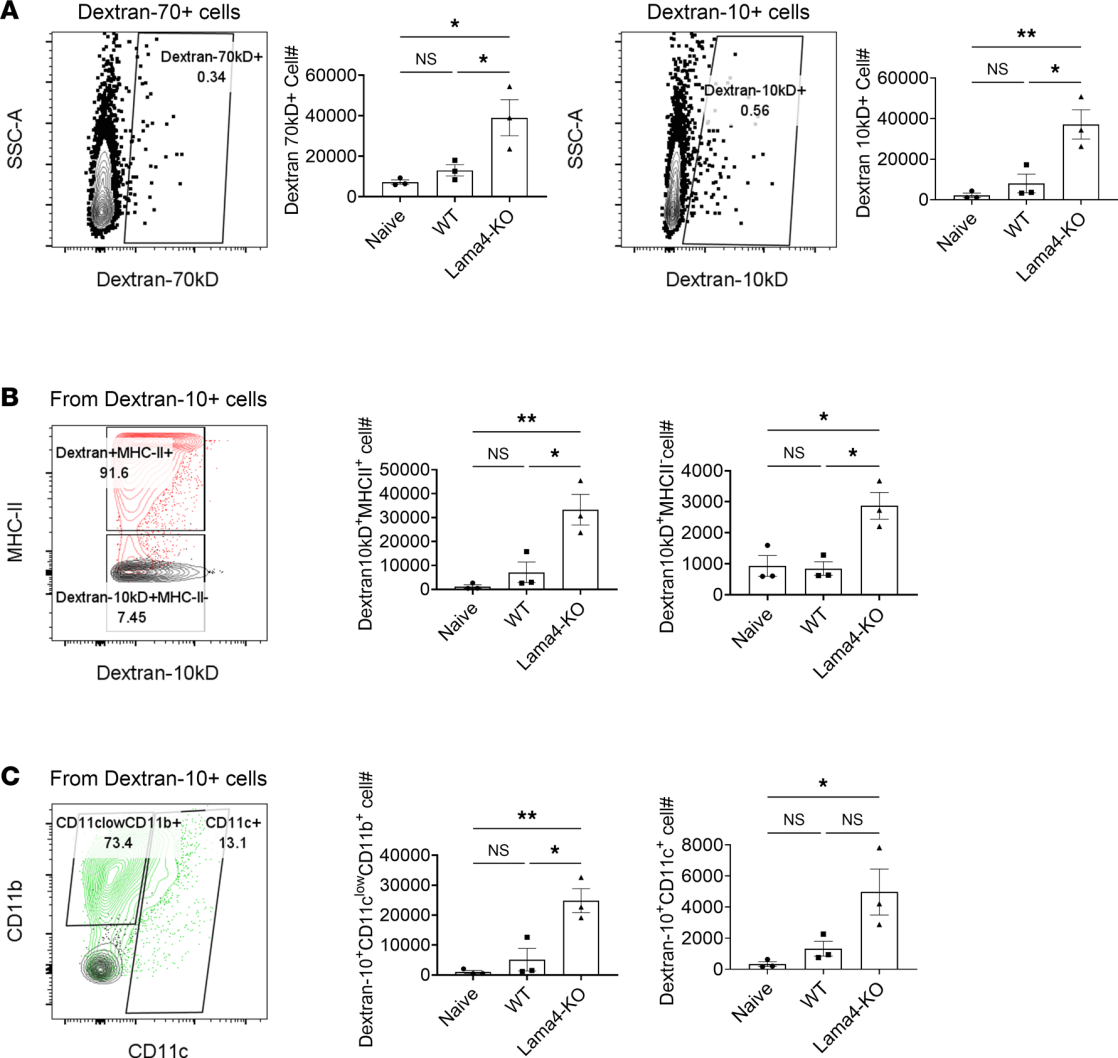
Laminins regulate conduit flow and antigen uptake after immunization. Mice were immunized with s.c. flank injection of IFA/OVA. Five days later, 10 kDa dextran-FITC and 70 kDa rhodamine B–dextran were injected at the tail base. Ninety minutes after dextran injection, inguinal dLNs were harvested for flow analysis. (**A**) Dextran-10^+^ and -70^+^ cells in dLNs. (**B**) Dextran-10^+^MHC II^+^ and dextran-10^+^MHC II^–^ cells. (**C**) Dextran-10^+^CD11c^lo^CD11b^+^ and CD11c^+^ myeloid subsets. Representative data of 2 independent experiments, 3 mice/group. Data presented as mean ± SEM. *P < 0.05; **P < 0.01 by 1-way ANOVA with Tukey’s multiple-comparison test.

We next asked whether Lama4 deficiency in FRCs would affect in vivo conduit functions under inflammatory conditions. WT and FRC-Lama4-KO mice were immunized with ovalbumin emulsified with incomplete Freund’s adjuvant (IFA/OVA) (5). Five days after immunization, all mouse strains displayed similarly enlarged dLNs, with 3-fold increases in LN cellularity (Supplemental Figure 4) relative to naive mice. No differences were observed between the laminin KOs and WT (Supplemental Figure 4), indicating that depleting laminins did not affect acute LN expansion. We then compared the flow of fluorescently labeled 10 kDa and 70 kDa dextrans (dextran-10 and -70, respectively) through the conduits of LNs acutely inflamed by IFA/OVA (5). Within 90 minutes of dextran injection, FRC-Lama4-KO inflamed LNs contained more dextran-10^+^ cells than WT (Figure 5A), indicating that depleting FRC Lama4 resulted in increased conduit flow of small molecules during acute immunization. Relative to WT dLNs, the FRC-Lama4-KO dLNs had more MHC II^+^dextran-10^+^ cells (Figure 5B). There was also an increase in dextran-10^+^CD11c^lo^CD11b^+^ monocytes/macrophages and dextran-10^+^CD11c^+^ DCs in the FRC-Lama4-KO inflamed dLNs, relative to WT controls (Figure 5C). These results indicated that conduit transport of antigens or antigen-presenting cells was enhanced after depletion of FRC Lama4. Overall, despite the decreased conduit flow under homeostatic conditions (Figure 2), depletion of FRC Lama4 augmented conduit flow and antigen uptake by the conduit network during acute immunization. These observations are commensurate with the more proinflammatory and immunogenic environment of the FRC-Lama4-KO LN, as we recently described (24).

### FRC transfer restores FRC-Lama4-KO LN impairments and allograft acceptance.

**Figure 6:**
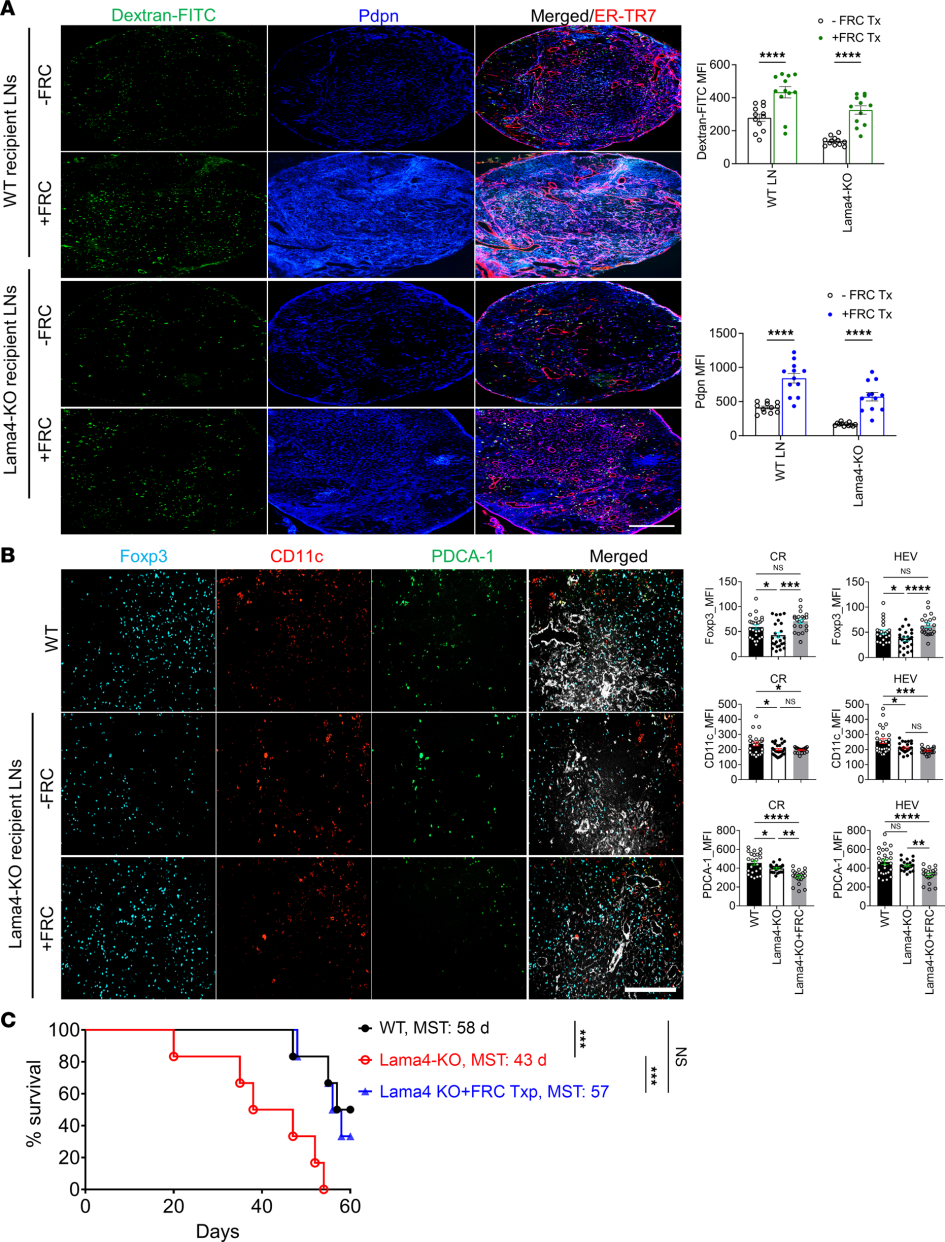
FRC transfer restores FRC-Lama4-KO lymph node impairments and allograft acceptance. WT FRCs (1 × 10^5^) were injected i.v. into WT or FRC-Lama4-KO mice weekly for 4 weeks. One week after the fourth dose, conduit systems were visualized 90 minutes after injecting 40 kDa dextran-FITC. (**A**) Left: Whole-mount scanning fluorescence images of LN cryosections from WT and FRC-Lama4-KO mice with and without FRC transfer. Scale bar: 500 μm. Right: Quantification of dextran-FITC and Pdpn in LNs. (**B**) FoxP3^+^ Tregs, CD11c^+^ cDCs, and Pdca-1^+^ pDCs in LNs. Scale bar: 100 μm. Data in **A** and **B** are representative of 3 independent experiments; 3 mice/group, 5 LNs/mouse, 3 sections/LN, 3–5 fields/section. (**C**) One week after the fourth dose of FRC, Lama4-KO mice received cardiac transplants from BALB/c mice, 250 μg anti-CD40L mAb i.v. on day 0, and weekly WT FRCs after transplantation (1 × 10^5^ FRCs/dose/week); 6 recipients/group. Allograft survival was monitored for 8 weeks. WT and FRC-Lama4-KO recipients without FRC transfer were used as controls. Data presented as mean ± SEM. *P < 0.05; **P < 0.01; ****P < 0.001; ****P < 0.0001 by 1-way ANOVA with Tukey’s multiple-comparison test (**A** and **B**) or 2-tailed log-rank (Mantel-Cox) test (**C**).

As FRC Lama4 contributed to the integrity of the fibroblastic network and conduits, we sought to ameliorate defects in FRC-Lama4–deficient mice by transferring healthy WT FRCs into them. We previously validated that FRCs can be transferred intravenously (i.v.), and that they home to and populate recipient LNs (14, 27). We found that after 4 weekly i.v. doses of FRCs (1 × 10^5^ cells/week), Pdpn and dextran-FITC signals in the FRC-Lama4-KO LNs increased to levels comparable to those of WT LNs (Figure 6A). This indicated that transplantation of ex vivo–expanded FRCs could recover defective FRCs and conduits caused by depleting laminins (Figure 6A). We previously found that FRC-Lama4-KO LNs had reduced HEVs, chemokines, and Tregs (24). We explored whether FRC adoptive transfer could reverse those defects. Notably, Foxp3^+^ Tregs, which were significantly lower in Lama4-KO than WT, increased to levels comparable to WT (Figure 6B). CCL21 and CXCL12, which are responsible for T cell migration in LNs, also recovered to the WT level (Supplemental Figure 5). However, such FRC transfer failed to recover the reduced cDCs and pDCs in FRC-Lama4-KO LNs (Figure 6B). FRC transfer also did not restore HEVs (Supplemental Figure 6). Taken together, these results indicated that transplantation of WT FRCs could recover the blunted Tregs and chemokines but could not reverse the reduced HEVs and DCs.

The recovery of LN structures and Tregs encouraged us to explore allograft acceptance after FRC transplantation in FRC-Lama4-KO mice. Cardiac allograft transplantation from BALB/c mice to WT and FRC-Lama4-KO recipients was performed along with a single low dose of immunosuppression with 250 μg anti-CD40L mAb i.v. immediately after transplantation. One group of FRC-Lama4-KO recipients received 4 doses of WT FRCs as described above prior to transplantation, and FRC transfers were continued weekly for 2 months after transplantation. Without FRC transfer, the FRC-Lama4-KO recipients had shorter allograft survival compared with WT (median survival time [MST] 42.5 vs. 60 days, *P* < 0.01) (Figure 6C), as previously reported (24). Notably, transferring WT FRCs prolonged allograft survival in FRC-Lama4-KO recipients, which was similar to that of WT recipients (MST 57 vs. 58 days, *P* = 0.31) (Figure 6C), indicating that FRC transfer restored allograft survival that was impaired by FRC Lama4 depletion. Taken together, these results demonstrated that FRC-derived laminins maintained the intact LN fibroblastic reticular network and conduit system, and transfer of expanded WT FRCs could partially rescue the defective FRC network, conduits, chemokines, and Tregs in Lama4-KO mice, and in turn restore allograft acceptance.

### FRCs interact with BECs and LECs through collagen and laminin signaling pathways.

**Figure 7:**
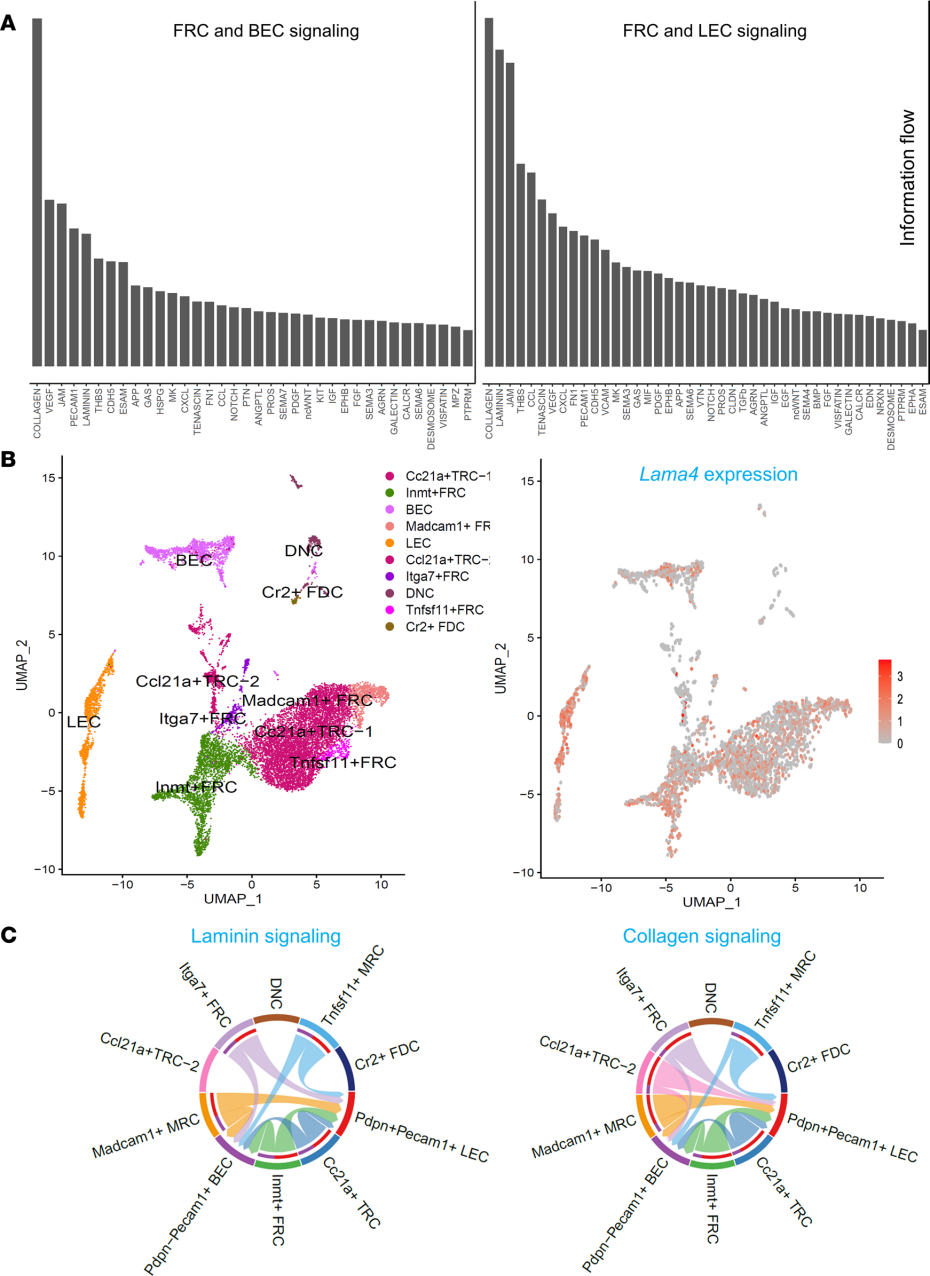
Lymph node stromal cell subsets crosstalk through laminin and collagen signaling pathways. (**A**) Bar plot summarizing the number of interactions of significant signaling pathways based on information flow. (**B**) LNSC subsets identified by specific markers (left) and *Lama4* gene expression (right) in these subsets. (**C**) Chord diagrams plotting signaling strength differences among different LNSC populations for the collagen and laminin pathways. Lines are the ligand-receptor interactions, and the relative thickness denotes strength. Outgoing signaling–targeted cell types represented by the color bars in the inner circles; outer color bars represent incoming signaling.

A recent study demonstrated that medullary FRC transdifferentiation drives lipomatosis and induces extensive vascular remodeling in aging human LNs. Such FRC dysregulation results in the dysfunction of immune structure in human LNs (28). This again emphasizes that FRC subsets sustain normal LN function by supporting the LN vasculature and the LN microenvironment as a whole. To quantify crosstalk among FRC and other LN stromal cells (LNSCs), and to specifically investigate the roles of conduit fibers, we defined the ligand-receptor communication flow among *Pecam1*^–^ *Pdpn*^+^ FRC subsets, blood endothelial cells (BECs), and LECs, using scRNA-seq. Collagen and laminin signaling pathways were ranked as the most remarkable signaling pathways (Figure 7A). FRCs were further classified into 2 *Ccl21^+^* T zone reticular cells (TRCs), *N*-methyltransferase^+^ (*Inmt^+^*) FRCs, *Madcam1*^+^ marginal reticular cells (MRCs), integrin α7^+^ (*Itga7*^+^) FRCs, tumor necrosis factor ligand superfamily member 11^+^ (*Tnfsf11^+^*) MRCs, *Pecam1^–^* *Pdpn^–^* (double negative cells, DNCs), and complement receptor 2^+^ (*CR2*^+^) FDCs according to the expression of distinctive genes (Figure 7B) (29, 30). The *Lama4* gene was widely expressed in FRC subsets, LECs, and BECs (Figure 7B). Further, we examined the signaling pathways among the FRC subsets and LECs and BECs. Collagen and laminin signaling pathways were widely involved in ECM stromal fiber interactions among these LNSCs (Figure 7C). A notable exception was FDCs, which had few interactions, commensurate with the data presented above (Figure 1) of the relative paucity of conduits in the B follicles. These results indicated that FRCs likely interact with LECs and BECs through collagen and laminin pathways that were critical for LN angiogenesis and architecture (21). These results suggested that LNSCs dynamically crosstalk with each other for sustaining the functional LN microenvironment.

## Discussion

Laminins belong to the ECM components enclosing the LN conduits, which channel soluble antigens to facilitate antigen sensing by resident immune cells (1, 2, 4, 5, 11). Global Lama4-KO mice develop hemorrhagic disease during the embryonic and neonatal periods, with extensive bleeding and deterioration of microvessel growth (23). Pharmacologic inhibition of laminins with mAbs can facilitate immunomodulation but lacks specificity and fails to identify the underlying mechanisms (20). Considering these limitations, we established the *Ccl19*-Cre/iDTR mouse strain to verify the contributions of FRCs to LN architecture and function. To further identify the regulatory effects of FRC-derived laminins and the underlying mechanisms, we established FRC-conditional forms of Lama4, Lama5, and LTβR strains. These strains helped us to define the roles of Lama4 and Lama5 in conduit development. Furthermore, we revealed that FRC-derived Lama4 maintains the phenotypic and functional integrity of FRCs, thereby ensuring the integrity of conduits and LTβR signaling. FRC-Lama4-regulated FRC proliferation and survival sustains FRC homeostasis and conduits. FRC-Lama4-KO mice demonstrated several defects in FRC, conduit, and LN structures and functions, each providing additional mechanistic evidence for how this conditional KO results in a proinflammatory and antitolerogenic environment.

Kelch et al. showed that Ki67-expressing cells in resting LNs are located very close to laminin^+^ conduits (18). The present study demonstrated that FRC Lama4 regulates Ki67 expression, and thus proliferation, of FRCs (Figure 4). We observed that the endogenous Lama4 maintains FRC proliferation and integrity both in vivo and in vitro, as supplementing Lama4 protein (laminin 411; see Methods) in FRC culture did not reverse the changes in primary FRC proliferation (Figure 4). This suggests a direct defect in proliferation. There are likely several additional indirect mechanisms underlying laminin-regulated FRC proliferation. We previously demonstrated that HEV numbers, CCL21, and CXCL12 increase in FRC-Lama5-KO LNs but decrease in FRC-Lama4-KO LNs (21, 24). HEVs and these chemokines provide the infrastructure for large-scale recruitment of lymphocytes from the blood into LNs (31). Consequently, the migration of immune cells from blood to LNs increases in FRC-Lama5-KO mice, but decreases in FRC-Lama4-KO mice relative to WT (21, 24). Immune cells in turn regulate FRC proliferation. For example, T cells and DCs can stimulate FRC expansion through LTβR signaling (32, 33), and DCs promote proliferation of FRCs through signal regulatory protein α (34). In experimental autoimmune encephalomyelitis and colitis, T cell–derived IL-17 promotes FRC proliferation by enhancing their metabolic fitness. IL-17R deficiency in FRCs leads to apoptosis and impaired expansion, in turn compromising humoral immune responses (35). CD11c^+^CD11b^+^ monocytes and DCs regulate VEGF production by FRCs through IL-1β, thereby affecting immunization-induced vascular-stromal proliferation (36). Since HEVs and conduits coordinately deliver cell or antigen in response to pathological stimuli, infections, or tumors, it is likely that the laminin-regulated LNSC proliferation is influenced by immune responses affecting the antigen/cell transportation infrastructure (8).

FRCs are attractive targets for addressing immunologic diseases and improving transplantation outcomes since they subsume many important functions related to antigen transport, antigen processing and presentation, and migration and positioning of DC and Tregs. LN transplantation is verified as a useful tool for replacing LNSCs for immunomodulation purposes. In the transferred LNs, the donor-derived LNSCs are retained but the immune cells are dominated by host origin (37). The LNSCs in transplanted LNs retain the capacity for recruiting recipient immune cells, T cell activation, and influencing DC-regulated Treg differentiation (37–39). This approach can also improve lymphedema by exchanges with the systemic circulation (39–41). Current FRC-based therapeutic investigations include administration of ex vivo–expanded FRCs and modulating FRC-derived molecules. Fletcher et al. demonstrated that administration of a single intraperitoneal (i.p.) dose of ex vivo–expanded FRCs after sepsis onset can reduce mortality in mice (42). Abdi and colleagues demonstrated that repetitive renal ischemia-reperfusion injury leads to fibrotic kidney dLNs and FRC senescence, whereas transfer of normal FRCs restores normal LN architecture and alleviates LN fibrosis (27). They further showed that systemic administration of FRCs can ameliorate LN fibrosis and promote tolerance induction after cardiac allografting. One recent study showed that assembly of FRCs into 3D spheroids enhances antigen-specific immune responses and antitumoral immunity in mice (43). More exploration is necessary to track the migration route and longevity of the transferred FRCs in the recipients. In the dLNs of mice with crescentic glomerulonephritis, an inflammatory disease featured by rapid deterioration of kidney function, activated FRCs help form an inflammatory milieu. Genetic depletion of FRCs or administration of anti-Pdpn antibodies ameliorates kidney injury (44). Hence, FRCs are involved in bidirectional regulation of immune responses — either pro- or antiinflammatory. How and to what extent the immune cell–nurturing fibroblastic niches steer immune responses, and how to therapeutically balance the immunosuppressive and immunostimulatory effects of FRCs, warrant further investigations. Furthermore, quality control of ex vivo–expanded FRCs is a challenge since they may lose their characteristic gene expression signatures over time. One study showed that ablation of MyD88 on FRCs promotes the secretion of IL-15, which maintains type 1 innate lymphoid cells (ILC1s) and, in turn, leads to viral clearance (45). However, the hyperactivation of ILC1s also results in severe intestinal inflammatory disease (45). To conquer these challenges, progress in novel reporter mice, advanced transcriptional analyses, and high-resolution histological analysis will identify new FRC subclusters and molecules (46). These in turn may contribute to fine-tuning immunomodulation for translational therapeutic strategies.

## Methods

### Mice.

CD45.2^+^ C57BL/6 (H-2^b^) mice were obtained from The Jackson Laboratory. To generate *Pdgfrb*-Cre^+/–^ × *Lama4^fl/fl^* (FRC-Lama4-KO) and *Pdgfrb*-Cre^+/–^ × *Lama5^fl/fl^* (FRC-Lama5-KO) mice, *Pdgfrb*-Cre^+/–^ mice provided by Ralf Adams (Max Planck Institute for Molecular Biomedicine, Muenster, Germany) (47) were crossed with *Lama4^fl/fl^* and *Lama5^fl/fl^* mice, respectively, and then backcrossed with C57BL/6 for 10 generations. In order to generate *Lama4^fl/fl^* mice, the *loxP* sequence (TATTGAAGCATATCGTATGTAATATGCTTCAATA) was inserted into the sites before and after exon 3 of the *Lama4* gene through CRISPR/Cas9 gene editing. *Lama5^fl/fl^* mice were a gift from Jeff Miner (Washington University School of Medicine, St. Louis, Missouri, USA) (48). Mice from these KO strains were healthy and fertile without any abnormal growth or development. Female mice between 8 and 12 weeks old were used for all experiments. *Ccl19*-Cre^+/–^ × *Ltbr^fl/fl^* and CCL19/iDTR strains were maintained in the Abdi lab. CCL19/iDTR mice were given daily 100 ng DT (MilliporeSigma, catalog 322326) i.p. to deplete CCL19^+^ FRCs. Two days after the fifth dose, the inguinal, brachial, and axillary LNs were collected. All mice were maintained under specific pathogen–free conditions.

### Reagents and antibodies.

Human recombinant laminin α4β1γ1 (laminin 411, catalog LN411) and 511 (catalog LN511) were from Biolamina. Dulbecco’s Modified Eagle’s Medium (DMEM, catalog 10-013-CV) was from Corning, and 10 kDa dextran-FITC (catalog D22910) and 40 kDa dextran-FITC (catalog D1845) were from Thermo Fisher Scientific. The PE Annexin V Apoptosis Detection Kit was purchased from BD Biosciences. Senescence β-Galactosidase Staining Kit was from Cell Signaling Technology. The antibody information is listed in Table 1.

### Cell preparations.

The cell mixture from LN enzymatic digestion (49) was cultured in complete DMEM supplemented with 10% fetal bovine serum (Gemini, catalog 900-108) and 1% penicillin-streptomycin (Thermo Fisher Scientific, catalog 15070063) for ex vivo expansion of mouse primary FRCs. The nonadherent cells were washed out after 48 hours and the remaining adherent cells were cultured in complete DMEM. Experiments were carried out on the fourth-passage cells, which contained greater than 90% CD31^–^Pdpn^+^ FRCs. For FRC transplantation, 1 × 10^5^ primary FRCs were filtered twice through a 70-μm strainer and then injected i.v. into WT, FRC-Lama4-KO, and FRC-Lama5-KO mice, once a week, for 3 weeks. One week after the third dose, the conduit system was visualized following the protocol described below.

### Visualization of conduit network.

Fluorescently labeled dextran was injected s.c. to aid in visualization of the conduit network. Various sizes of dextran-FITC (Oregon Green 488 Dextran, 10 kDa (Thermo Fisher Scientific, catalog D7170), dextran-FITC, 40 kDa (Thermo Fisher Scientific, catalog D1845), and rhodamine B–dextran, 70 kDa (Thermo Fisher Scientific, catalog D1841), doses (2.5 μg, 5 μg, and 10 μg), and time points (3, 5, 10, or 30 minutes) were assessed during preliminary studies (data not shown). Transit of 10 and 40 kDa dextran–FITC to LNs occurred within 2 minutes. A solution of 2.5 μg dextran-FITC (40 kDa or 10 kDa) in 20 μL PBS concomitant with harvesting at 5 minutes after injection provided a clear and distinguishable dextran-FITC fluorescence signal. The excised dLNs were placed in optimal cutting temperature (OCT) compound (Scigen Scientific, catalog 4583) on dry ice, and 6- or 40-μm cryosections were prepared for immunofluorescence microscopy.

### Immunization.

For acute immunization, mice were immunized via s.c. injection of 100 μL of IFA/OVA emulsion (Hooke Laboratories, catalog EK-0311) into the right and left flank (total of 200 mg OVA per mouse). Five days after immunization, mice were injected s.c. at the tail base with 100 μg 10 kDa dextran and 100 μg 70 kDa dextran in 40 μL PBS. Ninety minutes after injection, mice were euthanized for flow cytometry analysis (5).

### Flow cytometry.

LNs were disaggregated and passed through 70-μm sterile cell strainers (Thermo Fisher Scientific, catalog 223633548) to prepare single-cell suspensions. LNSCs were prepared by enzymatic digestion of freshly dissected LNs as described previously (50). Anti-CD16/anti-CD32 (eBioscience, catalog 14-0161-86) was used to block the Fc receptors prior to antibody staining, following the manufacturer’s instructions. Cells were then washed twice with FACS buffer (PBS with 0.5% w/v BSA) and fixed with 4% paraformaldehyde (Alfa Aesar, catalog J61899) for 10 minutes. Cells were fixed and permeabilized with a Foxp3 staining buffer set (Thermo Fisher Scientific, catalog 00-5523-00) for intracellular marker staining. Cells were run on an LSR Fortessa Cell Analyzer (BD Biosciences) and were analyzed with FlowJo software version 10.8 (Tree Star).

### scRNA-seq.

LNs were collected from three 12-week-old female C57BL/6 mice for enzymatic digestion. MojoSort Mouse CD45 Nanobeads (BioLegend, catalog 480028) were used to select for CD45^–^ LNSCs following the manufacturer’s instructions. Viable CD45^–^ LNSCs were then sorted for scRNA-seq, as described previously (24). Briefly, more than 2 × 10^4^ LNSCs were run on a 10× Chromium Controller (10× Genomics) to partition single cells into nanoliter-scale droplets containing uniquely barcoded beads. Sequencing libraries were prepared using a Chromium Single Cell 3′ Reagent Kit (v3 chemistry) (10× Genomics). The libraries were then sequenced on a NovaSeq 6000 sequencing system. Cells (4 × 10^3^ per sample) were captured on the 10× Chromium chip, and 5 × 10^4^ to 10 × 10^4^ reads/cell were obtained, with characterization of 2 × 10^4^ to 3 × 10^3^ transcripts/cell. Cell Ranger v3.1.0 (10× Genomics) was used to align sequences to the Ensembl mouse MM10 assembly. scRNA-seq analysis was performed using scRNA-seq data analysis software Seurat 3 (51). Cells with fewer than 200 or more than 5500 unique genes and more than 15% mitochondrial genes were excluded from subsequent steps to avoid dead cells or doublets. A total of 3329 cells were included for further analysis. A cell-cell communication network mediated by ligand-receptor interactions was obtained by applying CellChat (version 1.6.0) using the database CellChatDB of ligand-receptor pairs in mouse (52). Data were deposited in the NCBI Gene Expression Omnibus database (GEO GSE202068) (30).

### Immunofluorescence microscopy.

Immunofluorescence microscopy was conducted following a previously published protocol (21). In brief, mouse LNs were frozen in OCT (Sakura Finetek), and sectioned using a cryostat (Microm HM 550, Thermo Fisher Scientific). The cryosections were then fixed with cold acetone/methanol (1:1) solution for 5 minutes. Sections were then stained with primary antibodies, blocked with 10% secondary antibody host serum, incubated with secondary antibodies for 90 minutes, fixed with 4% paraformaldehyde in PBS for 5 minutes, quenched with 1% glycerol in PBS for 5 minutes, and mounted with Prolong Gold Antifade Mountant with or without DAPI (Thermo Fisher Scientific). Images were acquired using a Nikon Accu-Scope EXC-500 and EVOS FL Auto 2 (Thermo Fisher Scientific), and then analyzed using Volocity image analysis software (PerkinElmer). Mean fluorescence intensity (MFI) was quantified based on at least 3 independent experiments with 3 mice/group, 3 LNs/mouse, 3 sections/LN, and 3–5 fields/section.

### TEM.

TEM was conducted in the electron microscopy core imaging facility at the University of Maryland. Mouse LNs were fixed in 2% paraformaldehyde/1.5% glutaraldehyde/0.1 M sodium cacodylate (Electron Microscopy Sciences [EMS]) at 4°C for 16 hours, then in 1% osmium tetroxide/1.5% potassium ferricyanide at 4°C for 1 hour, followed by incubation in 1% tannic acid at room temperature for 45 minutes. Samples were dehydrated in increasing concentrations of ethanol solutions and then embedded in EMBed-812 (EMS, catalog 14900). Ultrathin resin sections (70 nm) were collected on formvar-coated slot grids after cutting on an ultramicrotome (UC7, Leica) using a DiATOME 45° diamond knife. Images were obtained using an FEI Tecnai T12 transmission electron microscope.

### Statistics.

Experiments were conducted in triplicate with at least 3 samples in each experiment. GraphPad Prism software v9 was used for generating figures. Results are presented as mean ± SEM. Two-group comparisons were analyzed using a Student’s *t* test for single variable differences. Multiple (>2) group comparisons were calculated using 1-way or 2-way ANOVA with Tukey’s multiple-comparison test. A *P* value of less than 0.05 was considered statistically significant.

### Study approval.

All animal experiments were performed in accordance with Institutional Animal Care and Use Committee approved protocols — Breeding Protocol for Transplant Tolerance and Lymphocyte Function Studies, no. 0821002; Experimental Protocol for Lymph Node Structure and Function in Tolerance: Role of Laminins, no. 1220001.

## Author contributions

JSB and LL conceived the study, designed experiments, interpreted the data, and wrote the manuscript. LL, LW, AK, and JZ conducted the majority of the study. The authorship order among the 4 co–first authors was assigned according to their contribution and commitment to this study. MWS, WP, TZ, ZM, YS, BM, VS, YSL, YX, XL, and XF contributed to experiments and the flow cytometry assay. YS performed the scRNA-seq data analysis. AK genotyped the mouse strains. AK and SJG revised the manuscript with constructive suggestions. RA revised the manuscript and supplied constructive suggestions on interpreting results.

## Supplementary Material

Supplemental data

## Figures and Tables

**Table 1:**
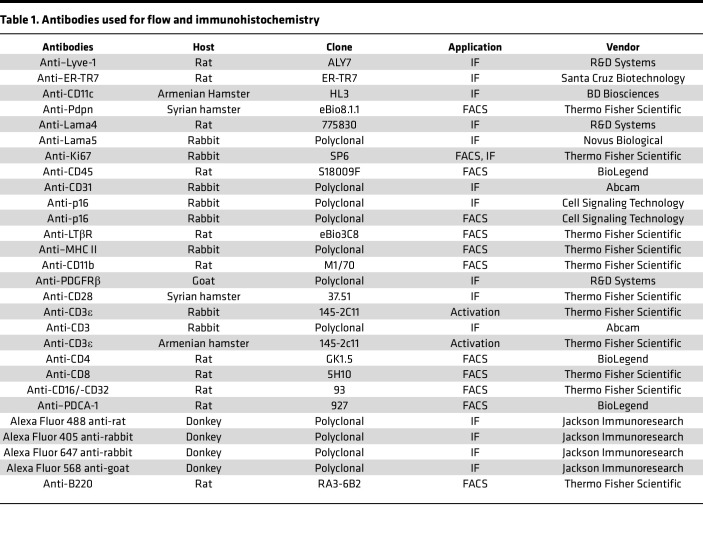
Antibodies used for flow and immunohistochemistry

## Version 1 04/24/2023

Electronic publication

## Version 2 03/18/2025

The GEO accession number was corrected in the Methods section
