# Supplementary material for: FRC transplantation restores lymph node conduit defects in laminin α4–deficient mice
Source: JCI Insight. 2023 Apr 24;8(8):e167816. doi: 10.1172/jci.insight.167816 (PMC10243809; doi:10.1172/jci.insight.167816)
Supplement: Supplemental data [file jciinsight-8-167816-s009.pdf]

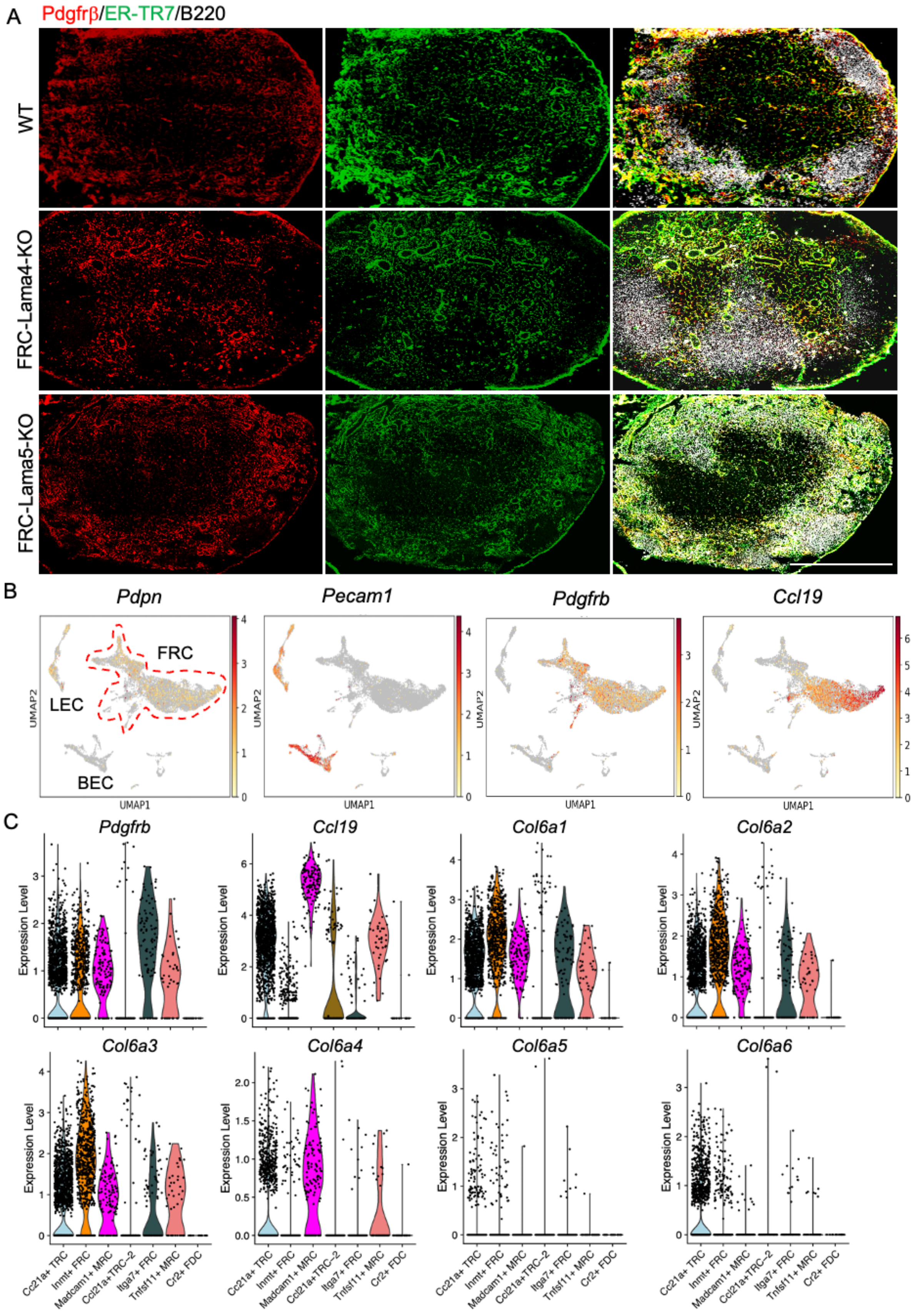
A
Pdgfrβ/ER-TR7/B220
WT
FRC-Lama4-KO
FRC-Lama5-KO
B
Pdpn
Pecam1
Pdgfrb
Ccl19
FRC
LEC
BEC
UMAP1
UMAP2
C
Pdgfrb
Ccl19
Col6a1
Col6a2
Col6a3
Col6a4
Col6a5
Col6a6
Expression Level
Cc21a+ TRC
Inmt+ FRC
Madcam1+ MRC
Ccl21a+TRC-2
Itga7+ FRC
Tnfsf11+ MRC
Cr2+ FDC

Figure S1. **Platelet-derived growth factor receptor β (PDGFRβ) is stably expressed by FRCs.** LNs were isolated from Pdgfrb-Cre$^{-/-}$ x Laminin α4$^{fl/fl}$ (WT), Pdgfrb-Cre$^{+/-}$ x Laminin α4$^{fl/fl}$ (FRC-Lama4-KO), and Pdgfrb-Cre$^{+/-}$ x Laminin α5$^{fl/fl}$ (FRC-Lama5-KO) mice. **A:** Whole mount scanning (20x) of 6 µm sections stained for Pdgfrβ, ER-TR7, B220, scale bar 500 µm. Representative of three independent experiments. 3 mice/group, 5 LNs/mouse, 3 sections/LN, 3-5 fields/section. **B**: *Pdpn, Pecam1, Pdgfrb,* and *Ccl19* gene expression in LN stromal cells, *Ccl19* and *Pdgfrb* are exclusively expressed by *Pdpn*$^{+}$*Pecam1*$^{-}$ FRCs. **C:** *Pdgfrb, CCL19, Pdpn, and Col6a1-Col6a6* expression in FRC subsets. FRCs were further classified into two *Ccl21*$^{+}$ T zone reticular cells (TRCs), *N-methyltransferase (Inmt)*$^{+}$ FRCs, *Madcam1*$^{+}$ marginal reticular cells (MRCs), *integrin α7* (*Itga7)*$^{+}$ FRCs, *tumor necrosis factor ligand superfamily member 11* (*Tnfsf11)*$^{+}$ MRCs, and *complement receptor 2 (CR2)*$^{+}$ FDCs according to the expression of distinctive genes. Representative of three independent experiments. 3 mice/group, 5 LNs/mouse, 3 sections/LN, 3-5 fields/section.

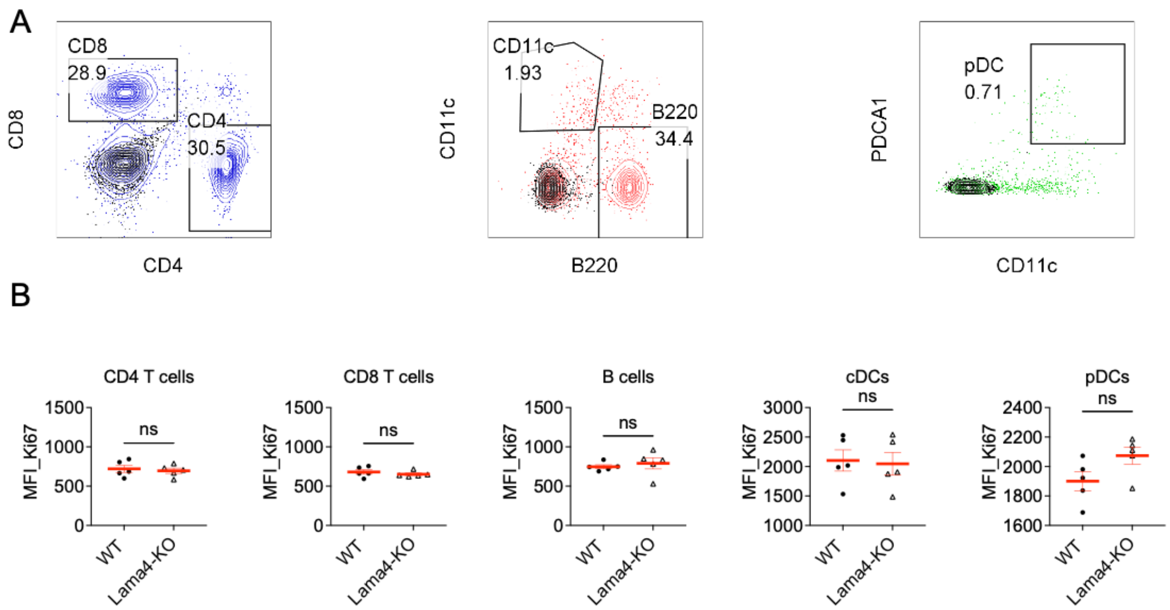

Figure S2. **Proliferation of freshly isolated leukocytes**. **A**: Gating of CD4 T cells, CD8 T cells, B cells, conventional dendritic cells (cDCs), and plasmacytoid DCs (pDCs) for flow cytometry. **B**: Ki67 intensity in these freshly isolated immune cells from WT and FRC-Lama4-KO LNs. Students' t-test for two groups comparison. Mean ± SEM.

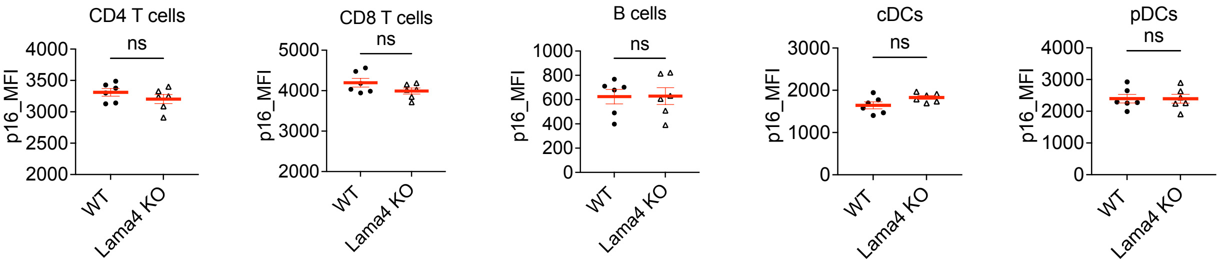

Figure S3. **Senescence of freshly isolated leukocytes**. Flow cytometry analysis of p16 expression in CD4 T cells, CD8 T cells, B cells, cDCs, and pDCs freshly isolated from WT, FRC-Lama4-KO, FRC-Lama5-KO LNs. Gating as in Fig. S2. Students' t-test for two groups comparison. Mean ± SEM.

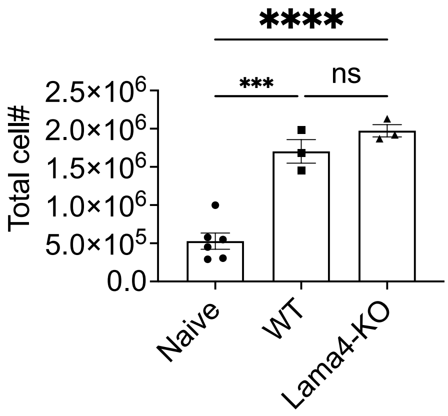

Figure S4: Mice were immunized in the flanks with IFA/OVA s.c.. Five days later, inguinal dLNs were harvested and total number of cells were quantified using flow cytometry. Representative data of two independent experiments, 3 mice/group. One-way ANOVA for multiple groups comparison. Mean ± SEM, ****$p<0.0001$.

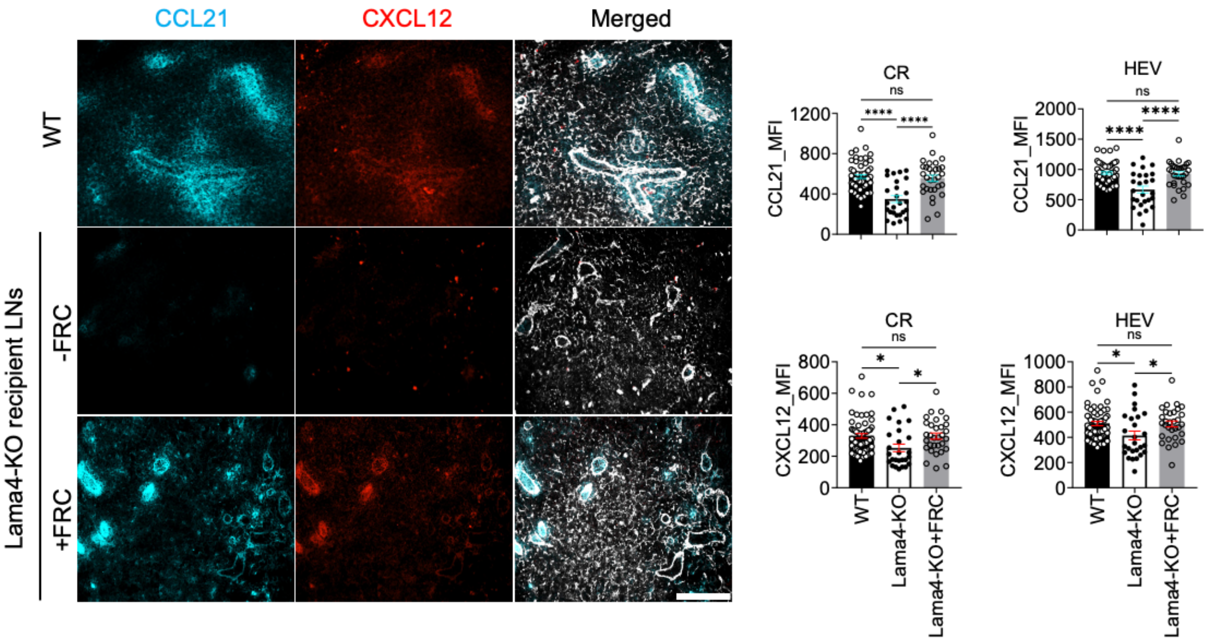

Figure S5: Transferring WT FRCs recovers chemokine distribution in FRFC-lama4-KO mice. WT FRCs were injected (i.v.) into FRC-Lama4-KO mice (10^5 FRCs/dose/week x 4 weeks). LNs from WT and FRC-Lama4-KO mice with or without FRC transfer were harvested for immunofluorescent staining for CCL21 and CXCL12. Scale bar 100 μm. Representative of three independent experiments. 3 mice/group, 5 LNs/mouse, 3 sections/LN, 3-5 fields/section. One-way ANOVA with Tukey's multiple comparisons test for multiple groups comparison. Mean ± SEM, *p < 0.05, ****p < 0.0001.

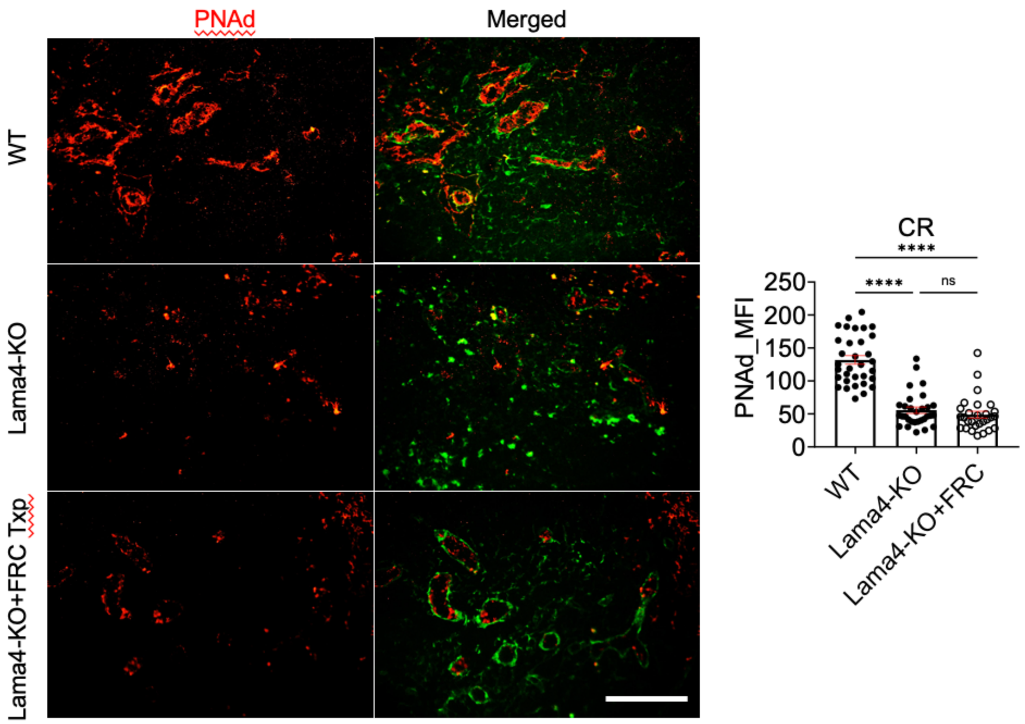

Figure S6. Transfer of WT FRCs did not rescue the defect of HEVs in FRC-Lama4-KO mice. 10^5 primary WT FRCs injected i.v. into WT and FRC-Lama4-KO mice, once a week for four weeks. One week after the 4th dose, LNs harvested. Left, cryosections stained for PNAd and ER-TR7. Right, quantification of PNAd intensity in cortical ridge (CR). One-way ANOVA for multiple groups comparison. Scale bar 100 um. Mean ± SEM, ****$p < 0.0001$.
